# Novosphingobium aeonii sp. nov., isolated from leaves of Aeonium decorum, is able to grow with polycyclic aromatic hydrocarbons

**DOI:** 10.1099/ijsem.0.007220

**Published:** 2026-06-23

**Authors:** Ana Segura, Lázaro Molina, Mafalda Domínguez, Félix Velando, Irene Hurtado, Pieter van Dillewijn, Zulema Udaondo

**Affiliations:** 1 Department of Environmental Microbiology and Biotechnology, Estación Experimental del Zaidín. CSIC, Granada, Spain; 2 Department of Microbial Biotechnology, Centro Nacional de Biotecnología, CSIC, Madrid, Spain

**Keywords:** 16S rRNA gene, *Aeonium*, average nucleotide identity (ANI), novel species, *Novosphingobium*, phyllosphere, phylogenomics

## Abstract

Atmospheric pollution poses a major threat to human health, causing millions of deaths worldwide each year. Green infrastructures may help mitigate the toxicity of airborne contaminants through the combined action of plants and their associated microbiota. Two bacterial isolates, I1^T^ and FS9, representing a novel species of the genus *Novosphingobium*, were obtained from the leaves of *Aeonium decorum*, a succulent plant native to the Canary Islands (Spain), based on their ability to grow using phenanthrene as the sole carbon source. The genomes of both isolates were sequenced using a hybrid approach combining short- and long-read technologies, resulting in four complete, circularized replicons per genome. A genus-wide comparative analysis including 400 quality-checked *Novosphingobium* genomes, among them 74 assemblies corresponding to type strains and the 2 novel isolates*,* revealed that the closest validly published species was represented by the type strain genome of *Novosphingobium lindaniclasticum* LE124^T^ (DSM 10700^T^=NBRC 16058^T^), which showed the highest ANI values with isolates I1^T^ and FS9, 86.60% and 86.58%, respectively, and dDDH values of 26.4%, supporting their assignment to a novel species. This analysis also identified four additional strains, previously deposited in public databases as *Novosphingobium* sp*.*, which fall within the ANI and genomic distance thresholds (≥95%) of the proposed species. Phylogenetic analysis based on 16S rRNA gene sequences and chemotaxonomic characterization further corroborated this taxonomic placement. We propose the name *Novosphingobium aeonii* sp. nov. for this novel species. The proposed type strain, *N. aeonii* I1^T^ (CET 31212^T^=DSM 119885^T^), has a genome size of 5.76 Mbp and a DNA G+C content of 64.76%.

## Data Summary

The authors confirm that all supporting data, codes and protocols have been provided within the article or in the supplementary information and material files.

## Introduction

The genus *Novosphingobium* is a member of the family *Erythrobacteraceae* within the class *Alphaproteobacteria*, with *Novosphingobium capsulatum* designated as the type species [1]. At present, 70 species with validly published names are recognized within the genus (https://www.bacterio.net/genus/novosphingobium). Originally established by Takeuchi *et al.* in 2001 following its reclassification from the genus *Sphingomonas* [2], *Novosphingobium* comprises Gram-negative, strictly aerobic, non-sporulating, rod-shaped bacteria, typically measuring 0.3–0.6 µm in width and 0.8–1.5 µm in length. Members of the genus usually form yellow-pigmented colonies and are characterized by the presence of glycosphingolipids in their cell envelope together with ubiquinone-10 as the major respiratory quinone, spermidine as the predominant polyamine and 2-hydroxy fatty acid C_14:0_ 2-OH as a major cellular fatty acid [2].

Species of *Novosphingobium* are typically environmental and have been isolated from diverse habitats, including soil, coastal and freshwater sediments [3,6], bog lakes [7,9], hot springs [10], activated sludge and wastewater treatment facilities [11,12] and bioremediation reactors treating contaminated groundwater [13,14]. In addition, several members of the genus have been reported in association with plants [15]. Owing to their remarkable metabolic versatility, many *Novosphingobium* strains are considered promising candidates for the bioremediation of contaminated water and soil, particularly due to their ability to degrade mono-, poly- and heterocyclic aromatic hydrocarbons [3,4, 16,19].

As part of our studies on the bioremediation of atmospheric contaminants, we aimed to isolate phenanthrene-growing strains from the phyllosphere of different plant species. Polycyclic aromatic hydrocarbons (PAHs) are ubiquitous atmospheric pollutants and major components of particulate matter (PM), many of which are toxic and carcinogenic. Because they have been found in PM, PAHs are nowadays regarded as re-emerging pollutants and are prioritized for elimination. Plants are capable of absorbing, adsorbing and metabolizing these compounds, and their associated microbiota can contribute to their transformation and elimination [20]. The phyllosphere represents an extensive interface for the deposition of airborne contaminants; however, the diversity and functional potential of phyllospheric bacteria remain comparatively understudied.

In this study, we report the isolates I1^T^ and FS9, as novel members of the genus *Novosphingobium* recovered from the leaf surface of the succulent plant *Aeonium decorum* Webb ex Bolle, a member of the family *Crassulaceae. Aeonium decorum*, first described in Bonplandia in 1859 [21], is native to the Canary Islands (Spain), where it typically grows on rocky surfaces and walls. Based on the results of our phylogenomic and comparative analyses, the isolates I1^T^ and FS9 are shown to represent a novel species within the genus *Novosphingobium*, most closely related to *Novosphingobium lindaniclasticum* [22] and *Novosphingobium resinovorum* [23].

## Methods

### Isolation of strains

Bacteria capable of degrading PAHs and belonging to the genus *Novosphingobium* were targeted, as members of this genus have been shown to exhibit a high capacity for the degradation of aromatic contaminants while colonizing diverse habitats under complex environmental conditions [24]. Samples were collected from adult plants purchased from a commercial nursery garden (Agrogojarviveros, Gójar, Granada, Spain). Leaves were excised and incubated in 10 ml M9 minimal medium with agitation for 16 h. Subsequently, the plant material was removed, and phenanthrene (1 mg) was added to the medium as the sole carbon source. The cultures were incubated at 30 °C with agitation (200 strokes/min) for 2 days. One millilitre of the culture was then transferred to a new flask with 10 ml of fresh M9 minimal medium supplemented with phenanthrene (1 mg) as the sole carbon source. This enrichment step was repeated twice under the same conditions. After the final enrichment, serial dilutions were plated onto Luria–Bertani (LB) agar plates to obtain single colonies. Ten yellow pigmented colonies, considered *Novosphingobium* precandidate strains, were selected and streaked onto fresh LB plates to obtain pure cultures.

For preliminary identification of the isolates, one colony from each pure culture was randomly selected and genomic DNA was extracted using the Wizard^®^ Genomic DNA Purification Kit (Promega) following the manufacturer’s instructions. Amplification of the 16S rRNA gene was performed using primers 16 S-27F (5′-AGAGTTTGATCMTGGCTCAG-3′) and 16 S-1492R (5′-TACGGYTACCTTGTTACGACTT-3′). PCR products were purified from agarose gels using the QIAquick Gel Extraction Kit (QIAGEN) following the manufacturer’s instructions and sequenced by Sanger sequencing (Genomic Service, Instituto de Parasitología y Biomedicina López-Neyra, CSIC-Granada, Spain). The resulting sequences were compared with 16S rRNA gene sequences available in the GenBank databases using blast+ [25,26]. Two yellow pigmented colonies identified as putative *Novosphingobium* spp. were selected for further analysis.

### DNA extraction, genome sequencing and genome assembly using a hybrid approach

Genomic DNA was extracted from pure cultures using the ZymoBIOMICS^™^ DNA Miniprep Kit (Zymo Research Corp., Irvine, CA, USA). DNA purity was assessed with a NanoDrop spectrophotometer by measuring the A260/A280 and A260/A230 ratios, while DNA concentration was determined using a Qubit Fluorometer v3.0 (Invitrogen) using the Qubit dsDNA BR Assay Kit. Purified DNA was aliquoted into two samples: one for MinION (Oxford Nanopore Technologies, Oxford, UK) and another for Illumina (San Diego, CA, USA) sequencing. Oxford Nanopore Technologies (ONT) sequencing libraries were prepared using a PCR-free method with multiplexed samples, employing the Rapid Barcoding Kit 24 V14 (SQK-RBK114.24). Sequencing of the barcoded DNA was conducted on a single R10.4.1 DNA flow cell using a MinION device (version Mk1B, ONT) over a 48-h period. For Illumina sequencing, libraries for the two isolates were sequenced using an Illumina NovaSeq6000^™^ sequencing platform (Illumina, Inc., San Diego, CA, USA). The Illumina library preparation and sequencing were performed by Novogene Co., Ltd. (UK), producing paired-end reads with a length of 2×150 bp.

Basecalling and demultiplexing of ONT reads, along with DNA adapter and barcode trimming, were performed using Dorado v0.8.0 (https://github.com/nanoporetech/dorado). ONT read quality was assessed with NanoPlot v1.43.0 [27]. Assembly of ONT reads was carried out using Flye v2.9.5-b180 [28] followed by one round of polishing with Medaka v2.0.0 (github.com/nanoporetech/medaka), and Homopolish v0.4.1 [29].

Pre- and post-processed Illumina paired-end reads quality was evaluated using FastQC v0.12.1 (https://www.bioinformatics.babraham.ac.uk/projects/fastqc/), and adapters were trimmed using fastp v0.23.4 [30]. Final polishing of the ONT assemblies was performed using Illumina paired-end reads and Polypolish v0.6.0 [31] followed by Pypolca v0.3.1 [32]. Assembly quality was assessed using CheckM v2 [33]. Plasmids replicons were confirmed using PLATON v1.7 using the accuracy mode [34]. The complete genome sequences of *Novosphingobium aeonii* isolates I1^T^ and FS9 were annotated using the National Center for Biotechnology Information (NCBI) Prokaryotic Genomes Annotation Pipeline [35].

Potential antimicrobial resistance genes were identified using the Center for Genomic Epidemiology webserver (https://cge.food.dtu.dk/services/ResFinder-EFSA/) that uses ResFinder 4.6.0 [36], and the Resistance Gene Identifier v6.0.3 from the Comprehensive Antibiotic Resistance Database (CARD) v3.3.0 [37]. Metabolic pathways were reconstructed using BlastKOALA against the KEGG (Kyoto Encyclopedia of Genes and Genomes) database [38] and Cluster of Orthologous Groups (COG) were assigned using eggNOG-mapper v2.1.12 [39]. Biosynthetic gene clusters were identified using antiSMASH 7.0.1 [40]. Prophage regions within the genomes were detected using the PHASTER web server [41], while genomic islands and integrative conjugative elements (ICEs) were identified using IslandViewer v4 [42] and ICEberg v.3.0 [43], respectively. Alignment and comparisons of the assemblies were performed using Mauve v.2.4.1 (https://darlinglab.org/mauve/user-guide/aligning.html). Circularized replicons were represented using Genovi software v0.2.16 [44].

### Taxonomic and phylogenomic analyses

The taxonomic position of both isolates was initially assessed using the Type (Strain) Genome Server (TYGS), maintained by the Leibniz Institute–Deutsche Sammlung von Mikroorganismen und Zellkulturen (DSMZ) [45]. The Genome-to-Genome Distance Calculator (GGDC) 3.0 was also used to perform *in silico* digital DNA–DNA hybridization (dDDH), applying a species threshold of 70% for species delineation [45].

Average nucleotide identity (ANI) values were calculated using the ANIm method [46] implemented in pyANI v0.3.0-alpha (https://github.com/widdowquinn/pyani), against a curated set of 398 *Novosphingobium* genomes retrieved from GenBank. This dataset included 74 genome assemblies corresponding to type strains of validly published *Novosphingobium* species for which genome sequences were available at the time of analysis (as of 5 September 2024). Pairwise alignments were generated using NUCmer from MUMmer4 [47]. A matrix of Mash distances was additionally calculated using Skani v0.2.2 [48], which is based on MinHash sketches [49].

The 16S rRNA gene sequences from the I1^T^ isolate were aligned using Megablast with the 16S ribosomal RNA (Bacteria and Archaea) database from the NCBI. Phylogenetic analyses were performed using a dataset of 249 nearly full-length (≥1,200 bp) 16S rRNA gene sequences extracted from the curated *Novosphingobium* genome dataset, with *Sphingomonas paucimobilis* included as an outgroup [1]. The 249 sequences were aligned using MAFFT v7.490 [50], and the maximum-likelihood phylogenetic tree was generated using IQ-TREE v2.3.6 [51]. The best-fit nucleotide substitution model was selected using ModelFinder [52], and the Kimura’s three-parameter model (K3P model) [53], with invariable sites plus Free Rate model (+I+R4), was used to construct the maximum-likelihood tree with ultrafast bootstraps with 1,000 replicates [54]. The resulting phylogenetic tree was visualized using the Interactive Tree of Life (iTOL) (v7.0) [55]. Pairwise ANI values were used to generate a distance matrix (1 − ANI), which was subjected to hierarchical clustering using Ward’s minimum variance method. The resulting dendrogram was exported in Newick format and visualized using the iTOL (v7.0) [55].

#### Physiology and chemotaxonomy

Cell morphology was examined by scanning electron microscopy after overnight growth in LB liquid medium at 30 °C with agitation (200 strokes min⁻¹). Observations were performed using a variable-pressure scanning electron microscope (Leo1430vp, Zeiss DSM 950, Supra40vp) at the Scientific Instrumentation Center of the University of Granada. Phenotypic characterization was conducted using the API 20NE system (bioMérieux, Durham, NC, USA) for isolates I1ᵀ and FS9, following the manufacturer’s instructions. Results were cross-checked using the BacDive API test finder database (https://bacdive.dsmz.de/api-test-finder; accessed 25 November 2024) [56].

Carbon, nitrogen and sulphur source utilization was evaluated using Biolog Phenotype MicroArray^™^ plates PM1, PM2A, PM3B and PM4A (Biolog Inc., Hayward, CA, USA). Plates were used as preconfigured substrate panels, and growth was assessed by turbidity measurements rather than dye-based respiration signals. PM1 and PM2A substrates (carbon sources) were prepared in 100 µl per well of M9 minimal medium supplemented with A9 (without carbon source). PM3B substrates (nitrogen sources) were prepared in M8 minimal medium lacking nitrogen and supplemented with A9 and glucose as a carbon source. PM4 substrates (sulphur sources) were prepared in M9 minimal medium lacking MgSO₄ and supplemented with glucose. Cells grown overnight in LB at 30 °C were washed twice with distilled water and inoculated to an initial OD₆₆₀ of 0.1. Plates were incubated at 30 °C with agitation (100 strokes min⁻¹). Optical density at 660 nm was measured at 0 and 48 h using a Varioskan LUX Multimode Microplate Reader (Thermo Fisher Scientific, Waltham, MA, USA) with SkanIt software v6.1. Growth was calculated as the difference between OD₆₆₀ values at 48 h and at inoculation (0 h). Growth was considered positive when OD₆₆₀ exceeded the corresponding negative control by at least 0.2 units. Experiments were performed in duplicate, and growth was only considered positive when both replicates met this criterion. Discordant wells were classified as negative.

Environmental tolerance assays were performed to determine the growth range of the isolates under varying salinity, pH and temperature conditions. Salt tolerance was assessed in M9 minimal medium supplemented with 20% glucose and 0.5% NaCl, with additional NaCl added to final concentrations of 4.3%, 7.8%, 15.1% and 29.7%. Cultures were inoculated to an initial OD₆₆₀ of 0.1 in 96-well microplates and incubated at 30 °C with continuous agitation in a Varioskan LUX reader. OD₆₆₀ was measured hourly for 25 h. Data represent the mean of five independent experiments. Growth was considered positive when OD₆₆₀ exceeded 0.5 after 25 h. Although OD₆₆₀ was recorded hourly to monitor growth dynamics, tolerance ranges were determined based on final OD values after 25 h of incubation.

Growth at different pH values was assessed under the same conditions using M9 minimal medium adjusted with phosphate buffers (NaH₂PO₄/Na₂HPO₄). Cells were grown overnight in LB, washed twice with distilled water and inoculated as described above. Growth was considered positive when OD₆₆₀ exceeded 0.5 after 25 h. Temperature tolerance was evaluated in 6-well plates using M9 minimal medium supplemented with glucose. Overnight cultures were inoculated to an initial OD₆₆₀ of 0.1 and incubated at 4, 20, 30, 37 and 42 °C. Optical density at 660 nm was measured after 24 h, and growth was considered positive when OD₆₆₀ exceeded 0.5.

Cellular fatty acid composition was performed at the Spanish Type Culture Collection (CECT, University of Valencia) using the MIDI Microbial Identification System (Sasser, 1990) with an Agilent 6850 gas chromatograph, applying the CLIN6 method (MIDI, 2008). Cells were grown in LB liquid medium at 30 °C for 24 h prior to fatty acid extraction.

Polar lipid and respiratory quinone analyses were carried out by DSMZ Services, Leibniz-Institut DSMZ – Deutsche Sammlung von Mikroorganismen und Zellkulturen GmbH, Braunschweig, Germany (provided in File S1, available in the online Supplementary Material).

## Results

### Morphological, physiological and phenotypic characterization of isolates I1^T^ and FS9

Cell morphology was examined after overnight growth in LB liquid medium at 30 °C for 24 h, using scanning electron microscopy. Cells of isolates I1^T^ and FS9 were rod-shaped, measuring ~1.33±0.15 µm in length and 0.49±0.02 µm in width for I1^T^ and 1.95±0.19 µm in length and 0.58±0.04 µm in width for FS9 (Figs S1 and S2). After 24 h of incubation on LB agar, colonies were yellow, with a smooth and glossy surface. These morphological features are consistent with those reported for other members of the genus *Novosphingobium* [57]. Both isolates grew on LB medium and on M9 minimal medium supplemented with glucose as carbon source. However, during liquid growth, strain FS9 consistently formed visible aggregates, which occasionally resulted in lower optical density readings in microplate-based assays. Physiological growth ranges were determined using defined minimal media. Growth was observed at temperatures between 20 and 37 °C, with optimal growth at 37 °C. Growth (defined as OD₆₆₀≥0.5 after 25 h of incubation) occurred at NaCl concentrations ranging from 0.5 to 7.8%, with optimal growth at 0.5% NaCl. The strains grew at pH values between 6 and 8, with an optimal pH of 7. These physiological properties are in agreement with those described for other *Novosphingobium* species [57].

The cellular fatty acid compositions of isolates I1ᵀ and FS9 are shown in Table 1. In both isolates, the predominant fatty acids were summed feature 8 (C_18:1_ ω7c and/or C_18:1_ ω6c), accounting for 55.4% of the total fatty acids in isolate I1ᵀ and 59.6% in isolate FS9. Other abundant fatty acids included summed feature 3 (C_16:1_ ω7c and/or C_16:1_ ω6c) and C_16:0_. Hydroxylated fatty acids C_14:0_ 2-OH and C_16:0_ 2-OH were also detected at notable levels. The presence of 2-hydroxy fatty acids is consistent with the occurrence of glycosphingolipids in the outer membrane, a chemotaxonomic hallmark of members of the family *Sphingomonadaceae*. In agreement with this, polar lipid analysis of the type strain I1ᵀ (DSM 119885ᵀ) revealed the presence of sphingoglycolipid, together with diphosphatidylglycerol, phosphatidylcholine, phosphatidylethanolamine, phosphatidyl-N-monomethylethanolamine, phosphatidyl-N,N-dimethylethanolamine, phosphatidylglycerol and an aminophospholipid. In addition, the major respiratory quinone of the type strain was Q-10, with minor amounts of Q-9. Overall, the fatty acid profiles of the two isolates were similar to those reported for *Novosphingobium guangzhouense* SA925ᵀ [58] and other *Novosphingobium* species, although differences in relative abundances were observed. Unsaturated C_18:1_ (summed feature 8) and C_16:1_ (summed feature 3) are typically the most abundant fatty acids in type strains of the genus [3,58, 59]. The predominance of these unsaturated fatty acids together with the presence of 2-hydroxy fatty acids supports the assignment of isolates I1ᵀ and FS9 to the genus *Novosphingobium* [57].

**Table 1.** Cellular fatty acid compositions (%) of isolates I1^T^, FS9 and other *Novosphingobium* species

	I1^T^	FS9	SA925^T^	LE124^T^	US6-1^T^	IK01^T^	UCM-28^T^
**Hydroxy fatty acid**							
C_12:0_ 2-OH	–	–	–	–	0.8	–	0.1
C_13:0_ 2-OH	–	–	–	–	–	–	0.3
C_14:0_ 2-OH	4.2	5.3	5.8	9.4	19.7	11.7	11.8
C_15:0_ 2-OH	–	–	0.2	–	0.3	–	1.7
C_16:0_ 2-OH	4.2	5.0	3.4	2.1	2.5	–	0.2
C_16:1_ 2-OH	–	–	–	–	–	–	0.4
iso-C_16:0_ 2-OH	–	–	–	–	–	–	0.5
**Saturated fatty acid**							
C_12:0_	–	–	0.1	0.1	–	–	–
C_14:0_	0.6	–	0.7	1	0.6	1.9	0.1
C_16:0_	12.9	9.9	11.9	6.7	1	5.2	3.9
C_17:0_	–	–	–	–	–	0.8	0.5
C_18:0_	0.8	0.8	0.5	0.3	–	1.5	0.3
C_19:0_ cyclo ω8*c*	–	–	–	–	–	6.5	0.6
**Unsaturated fatty acid**							
C_15 :1_ ω6*c*	–	–	–	–	–	–	0.2
C_16:1_ ω5*c*	2.5	3.1	2.9	3.3	–	–	1.2
iso-C_16:1_ h	–	–	–	–	–	–	0.2
C_16:1_ ω7*c*	–	–	–	–	–	–	–
C_16:1_*	–	–	–	–	8.8	–	–
C_17:1_ ω6*c*	1.7	1.8	1	0.9	–	1.8	4.7
C_17:1_ ω8*c*	–	–	0.1	0.9	–	–	0.7
C_17:1_**	–	–	–	–	2	–	
iso-C_18:1_ h		–	–	–	–	–	0.6
C_18:1_ ω5*c*	1.7	–	0.7	–	–	–	0.6
C_18:1_ ω7*c*	–	–	59.7	–	–	–	
C_18:1_ ω7*c* 11-methyl	3.9	1.4			–	–	–
**Summed feature 3**	12.2	13.0	10.7	19.9		12.8	24.9
**Summed feature 8**	55.4	59.6	–	49.1	64.0***	57.8	46.3
**Summed feature 9**	–	–	–	–	–	–	0.3

Isolates: I1T: *N. aeonii* I1T; FS9, *N. aeonii* FS9; Type strains: SA925T, *N. guangzhouense* SA925T; LE124T, *N. lindaniclasticum* LE124T; US6-1T, *Novosphingobium pentaromaticivorans* US6-1T; IK01T, *Novosphingobium pituita* IK01T; UCM28T, *Novosphingobium flavum* UCM-28T. Summed features contain one or more of the following fatty acids: summed feature 3 (C_16:1_ ω7c and/or C_16:1_ ω6c); summed feature 8 (C_18:1_ ω7c and/or C_18:1_ ω6c); summed feature 9 (iso-C_17:1_ ω9c and/or C_16:0_ 10-methyl). * includes C_16:1_ ω5c and/or C_16:1_ ω7c; ** includes C_17:1_ ω6c and/or C_17:1_ ω8c; *** includes C_18:1_ ω 5 c. The cis isomer is indicated by ‘*C*’.

Both isolates were identified as Gram-negative, oxidase-positive and strictly aerobic. A comprehensive summary of the distinguishing morphological and physiological features of isolates I1^T^ and FS9 and those of closely related *Novosphingobium* species is presented in Table 2.

**Table 2.** Morphological and physiological properties of isolates I1^T^ and FS9 and other closely related *Novosphingobium* species

Phenotypic characteristic/test	I1^T^	FS9	KF1	US6-1^T^	28**^T^**
**Growth in LB broth**	+	+*			
**Colony morphology**	Rounded, glossy, yellow	Rounded, glossy, yellow			
**Temperature range, °C; optimum**	20–37; 37	20–37; 37			
**pH range, % w/v; optimum**	6–8; 7	6–8; 7			
**NaCl range (% w/v): optimum**	0.5–7.8; 0.5	0.5–7.8; 0.5			
**API20NE**					
**Nitrate reduction (NO_3_**)	Negative	Negative	Negative	Negative	Positive
***β*-Galactosidase (PNG**)	Positive	Positive	Positive	Negative	Positive
**l-Arabinose, assimilation (ARA**)	Positive	Positive	Positive	Negative	Positive
**d-Mannose, assimilation (MNE**)	Positive	Positive	Negative	Negative	Positive
***N*-Acetyl-glucosamine, assimilation (NAG**)	Positive	Positive	Positive	Negative	Positive
**Gluconate, assimilation (GNT**)	Negative	Negative	Negative	Negative	Positive
**Capric acid, assimilation (CAP**)	Negative	Negative	Positive	Negative	Negative
**Malic acid, assimilation (MLT**)	Positive	Positive	Positive	Negative	Positive
**Citrate, assimilation (CIT**)	Positive	Positive	Positive	Negative	Negative
**Phenylacetic acid, assimilation (PAC**)	Negative	Negative	Negative	Positive	Negative

Growth ranges and optimal growth conditions for temperature, pH and NaCl concentration are indicated. For API 20NE tests, only differential results among the compared strains are shown. API 20NE results for *N. resinovorum* KF1, *Novosphingobium pentaromaticivorans* US6-1T and *N. capsulatum* 28T were obtained from the BacDive API test finder database [56].

* Strain FS9 forms visible aggregates during growth in liquid medium.

The ability of both *N. aeonii* isolates to grow on substrates other than phenanthrene as sole carbon, nitrogen and sulphur source was assessed using high-throughput phenotypic analysis. In total, 190 compounds were tested as sole carbon sources, 95 as sole nitrogen sources and 35 as sole sulphur sources. Both strains efficiently metabolized simple sugars, such as glucose, fructose, galactose, xylose and arabinose; disaccharides, including lactose, maltose and cellobiose; and oligosaccharides, such as maltotriose, palatinose and gentiobiose. Several amino acids, peptides and organic acids also supported the growth of at least one of the isolates. A preference for the utilization of sugars as a carbon source was observed. Both isolates also demonstrated a high versatility in nitrogen source utilization, with a preference for certain amino acids (Gln, His, Ala, Met, Arg, Asp, Pro, Ser) and dipeptides (Ala-Asp, Ala-Glu, Ala-Gly, Ala-Leu, Gly-Asn, Gly-Gln, Gly-Glu). Ornithine, agmatine and urea also supported a robust growth in both isolates. Sulphur utilization was more restrictive, with sodium sulphate and sodium thiophosphate being the preferred sulphur sources. Notably, although both isolates tested urease-negative in the API 20NE assay, their growth on urea in the Biolog system is consistent with the presence of alternative, urease-independent pathways for nitrogen acquisition as has been documented in *Sphingomonadaceae* [60,61]. The broad utilization of simple sugars and amino acids observed in the PM assays is consistent with the genomic enrichment of COG categories related to amino acid metabolism and carbohydrate transport. However, detailed genotype–phenotype associations were not further explored, as functional validation was beyond the scope of this taxonomic study.

These analyses showed that *N. aeonii* isolates were able to utilize a broad range of substrates. Assimilation of glucose, arabinose, maltose and malic acid, previously observed in API 20NE tests, was confirmed by positive growth of both strains in these substrates. However, assimilation of mannose, citric and malic acid was confirmed only for strain I1^T^.

As noted above, strain FS9 has a tendency to form aggregates, which resulted in consistently lower optical density values and greater variability between replicates in microplate-based assays compared with strain I1^T^. Consequently, highly conservative criteria were applied for positive growth, ensuring that all reported positive results are robust and reproducible, while negative results should be interpreted with caution. Overall, the type strain I1^T^ was able to utilize a wide range of carbon and nitrogen sources, whereas sulphur utilization was more limited.

**Fig. 1.**
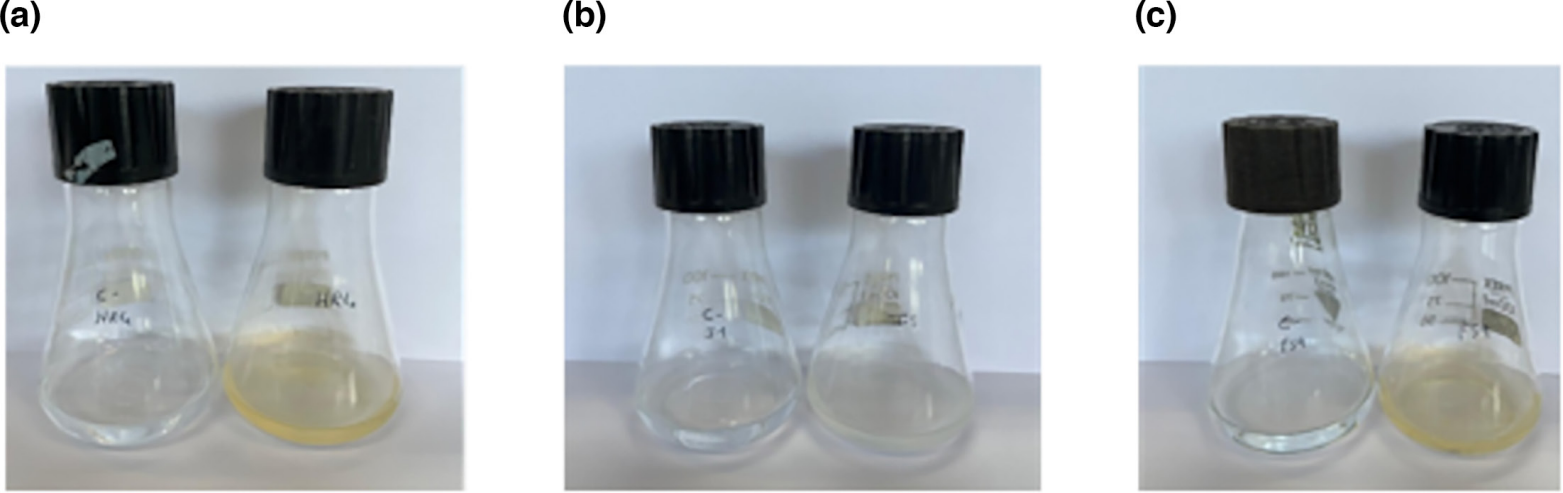
Cultures of *N. resinovorum* HR1a (**a**), *N. aeonii* I1ᵀ (**b**) and *N. aeonii* FS9 (**c**) after 3 days of incubation in M9 minimal medium without a carbon source (left flasks) or with phenanthrene as the sole carbon source (right flasks).

Both isolates were originally obtained based on their ability to grow using phenanthrene as the sole carbon source. However, clear phenotypic differences were observed during growth on this compound. Cultures of *N. aeonii* FS9 developed a pink colouration during growth on phenanthrene, whereas no colour change was observed for isolate I1ᵀ. A similar colouration has previously been reported during phenanthrene degradation by *N. resinovorum* HR1a and was associated with the transient accumulation of 1-hydroxy-2-naphthoic acid [6] (Fig. 1). These observations suggest differences in phenanthrene transformation processes between the two isolates. Genomic analyses identified several genes putatively encoding dioxygenases potentially involved in the initial steps of aromatic compound degradation. Although both genomes encode ring-hydroxylating dioxygenase components typically associated with the initial steps of aromatic compound degradation, the presence of these genes alone does not confirm the existence of a complete phenanthrene catabolic pathway. The specific metabolic route and the functional contribution of these genes to phenanthrene degradation in *N. aeonii*, therefore, require experimental validation.

### Main genomic features of isolates I1^T^ and FS9

Hybrid genome assembly resulted in four complete circular replicons for both isolates. CheckM analysis indicated 100% genome completeness, with low contamination values (1.84% for I1ᵀ and 2.09% for FS9), confirming high assembly quality.

**Fig. 2.**
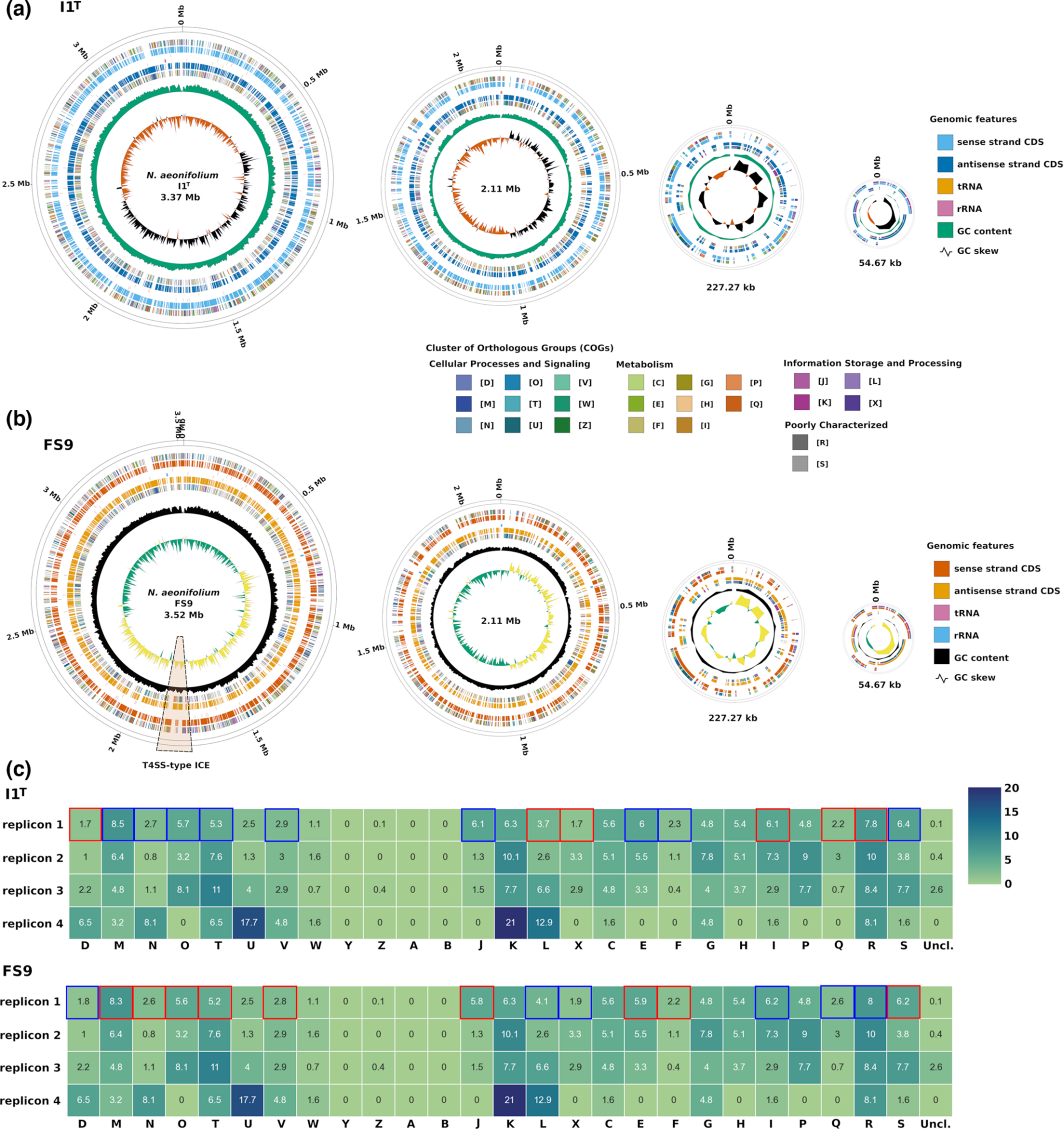
Circular genome maps and functional annotation for *N. aeonii* isolates I1^T^ and FS9. (**a**) Circularized replicons for isolate I1^T^: replicon 1 (3.37 Mb), replicon 2 (2.11 Mb), replicon 3 (227.27 kb) and replicon 4 (54.67 kb). Genomic features are annotated in the outermost rings, including the functional annotation of the coding sequences using COGs, sense strand coding sequences (CDS), antisense strand CDS, tRNA, rRNA, G+C content and GC skew. (**b**) Circularized replicons for isolate FS9: replicon 1 (3.52 Mb), replicon 2 (2.11 Mb), replicon 3 (227.27 kb) and replicon 4 (54.67 kb). Annotation follows the same scheme as in (**a**). (**c**) Heatmap showing the distribution of COG functional category frequencies as percentages across the four replicons of isolates I1^T^ and FS9. The rows represent the replicons, and the columns represent functional categories. The colour intensity reflects the percentage of proteins assigned to each COG category for each replicon, with darker shades indicating higher counts. Unclassified (Uncl.). COG functional categories: A, RNA processing and modification; B, chromatin structure and dynamics; C, energy production and conversion; D, cell cycle control, cell division, chromosome partitioning; E, amino acid transport and metabolism; F, nucleotide transport and metabolism; G, carbohydrate transport and metabolism; H, coenzyme transport and metabolism; I, lipid transport and metabolism; J, translation, ribosomal structure and biogenesis; K, transcription; L, replication, recombination and repair; M, cell wall/membrane/envelope biogenesis; N, cell motility; O, posttranslational modification, protein turnover, chaperones; P, inorganic ion transport and metabolism; Q, secondary metabolites biosynthesis, transport and catabolism; R, general function prediction only; S, function unknown; T, signal transduction mechanisms; U, intracellular trafficking, secretion, and vesicular transport; V, defence mechanisms; W, extracellular structures; X, mobilome: prophages, transposons; Y, nuclear structure; Z, cytoskeleton.

The complete genome of isolate I1ᵀ comprises 5.76 Mb, with a DNA G+C content of 64.76%, and includes four circular replicons, two of which were identified as plasmids (Fig. 2a, Table 3). The most notable genomic difference between the two isolates was observed in the main chromosomal replicon, which was ~158 kb larger in isolate FS9. This region was identified as an ICE encoding a type IV secretion system (Fig. 2b). ICEs are chromosomally integrated mobile genetic elements capable of horizontal transfer and frequently carry accessory genes that may confer ecological or adaptive advantages to their host. However, unlike plasmids, ICEs are stably integrated into the host chromosome and encode the genetic machinery required for their integration and excision [62]. The ICE identified in FS9 shows similarity to elements previously described in *N. resinovorum* HR1a, where related regions have been associated with genes involved in the transformation of polycyclic aromatic hydrocarbons [62]. While this finding is consistent with the phenotypic differences observed between isolates I1ᵀ and FS9 during growth on phenanthrene, the specific functional roles of ICE-associated genes in *N. aeonii* require further experimental confirmation.

**Table 3.** Features of the complete genome sequences of isolates I1^T^ and FS9

Isolate	Size (bp)	No. of complete replicons	%GC	N50	Coding ratio	Coverage	Total CDS	NCBI accessions
I1^T^	5,761,656	4	64.76	3365709	0.91	>×200	5,144	CP176832-35
FS9	5,920,010	4	64.67	3524063	0.91	>×200	5,315	CP176828-31

CDS, coding sequences; %GC, percentage of guanine (G) and cytosine (C) bases in the total DNA molecules.

Both genomes contain at least one gene putatively encoding a naphthalene/biphenyl dioxygenase, which may participate in the initial oxidation of aromatic substrates. Dioxygenases are known to display broad substrate specificity [63]. However, the involvement of this particular enzyme in phenanthrene degradation would need to be demonstrated experimentally before a specific role in PAH degradation can be assigned in strain I1ᵀ.

Functional annotation based on COG classification revealed similar profiles for both isolates. The most represented categories included ‘Amino Acid Metabolism and Transport’ (E), with 372 and 381 proteins for the I1^T^ and FS9 isolates, respectively, followed by ‘Transcription’ (K), with 368 and 379 assigned proteins. Other highly represented categories included ‘Inorganic Ion Transport and Metabolism’ (P), with 354 and 364 assigned proteins; ‘Energy Production and Conversion’ (C), with 316 and 331 assigned proteins; and ‘Cell Wall/Membrane/Envelope Biogenesis’ (M), with 299 and 303 assigned proteins and ‘Lipid Metabolism’ (I), with 78 and 283 assigned proteins for the I1^T^ and FS9 isolates, respectively. Additionally, the ‘Function Unknown’ (S) category was the most abundant overall, with 941 and 966 proteins assigned to this category for the I1^T^ and FS9 isolates, respectively. Accordingly, many amino acids and dipeptides are included among the N sources that supported the growth of the strains. Given the genomic similarities between both strains, the physiological discrepancies observed in the utilization of carbon and nitrogen sources were probably related to the flocculation of strain FS9 rather than to the real metabolic capacities of the strains.

Genome-wide screening for antimicrobial resistance genes using CARD and ResFinder identified only intrinsic efflux-related genes commonly present in environmental bacteria, with no acquired or clinically relevant antibiotic resistance determinants detected in either isolate. Secondary metabolite biosynthetic potential was explored using antiSMASH v7.0.1. Both genomes encoded a limited number of predicted biosynthetic gene clusters, including terpene-associated regions, a type III polyketide synthase (T3PKS), a ranthipeptide (RiPP-like) cluster, a hydrogen cyanide biosynthetic cluster and an ectoine biosynthetic gene cluster. The ectoine cluster was identified with medium similarity to known ectoine biosynthetic pathways, consistent with the observed tolerance to moderate salinity. The terpene-associated regions showed low similarity to characterized carotenoid clusters, suggesting potential pigment biosynthesis.

The main genome features of the isolates I1^T^ and FS9 are summarized in Table 3. Overall, the genomic architecture of both isolates is consistent with that of other *Novosphingobium* species, which typically harbour multiple replicons and a high proportion of genes involved in carbohydrate and amino acid metabolism. The presence of an ICE-like region in FS9 suggests potential ecological diversification related to aromatic compound transformation, although functional roles remain to be validated experimentally. In addition, both genomes encode complete pathways for central carbohydrate metabolism, amino acid utilization and inorganic ion transport, which is consistent with the broad phenotypic substrate range observed in the PM assays. Together, these genomic traits support the ecological versatility of *N. aeonii* while remaining aligned with the metabolic profiles characteristic of the genus.

### Taxonomic placement and genomic comparisons of novel *Novosphingobium* isolates

The TYGS, maintained by the Leibniz Institute DSMZ and linked to the List of Prokaryotic names with Standing in Nomenclature [45], was used to identify closely related type strains and to obtain an initial taxonomic placement of the two isolates. TYGS analyses assigned both isolates to the genus *Novosphingobium* and suggested their potential designation as a novel species (File S1). *In silico* dDDH values were calculated using formula 2 of the GGDC (https://ggdc.dsmz.de/distcalc2.php, accessed on 11 December 2024) [64,65]. For both isolates, dDDH values ranged from 20 to 26% (distances ranging from 0.16 to 0.22) when compared with the genomes of 74 *Novosphingobium* type strains. These values are below the 70% species delineation threshold, supporting the classification of the isolates as members of a novel genomospecies within the genus *Novosphingobium*, distinct from all currently described species (Tables S1 and S2).

Average nucleotide identity based on Mummer (ANIm) was calculated using pyANI [66], against a curated dataset of 398 *Novosphingobium* genomes, including 74 type strain genome assemblies. The closest type strain identified was *N. lindaniclasticum* LE124^T^ (CCM 7976^T^; DSM 25409^T^) [22], with ANI values of 86.60% and 86.58% for isolates I1^T^ and FS9, respectively. These values fall below the accepted species threshold of 95% ANI (see Table S3). In addition, ANIm analysis identified four additional strains deposited in public databases with ANI values above the species threshold (≥95%) among themselves and with our isolates (assembly IDs: GCA_035658495.1, GCA_004341305.1, GCA_001298105.1 and GCA_009360525.1) (Tables 4 and S3). Hierarchical clustering based on ANI values grouped these genomes together with isolates I1^T^ and FS9 in a distinct cluster, further supporting their assignment to the same novel species (Fig. S3). These strains, therefore, represent additional members of the same novel species and should be considered for reclassification accordingly.

**Table 4.** Table with the information on the four other *N. aeonii* strains

Assembly ID	Strain name	Isolation source	Isolation date
GCA_001298105.1	*Novosphingobium* sp. ST904	Rhizosphere soil of *Acer pseudoplatanus*	2015
GCA_004341305.1	*Novosphingobium* sp. ST904	No information available	2019
GCA_009360525.1	*Novosphingobium* sp. RQ_Bin_7	Activated sludge of kraft-pulp mill effluents	2019
GCA_035658495.1	*Novosphingobium* sp. RL4	Rhizosphere soil of *Rheum palmatum* L.	2024

**Fig. 3.**
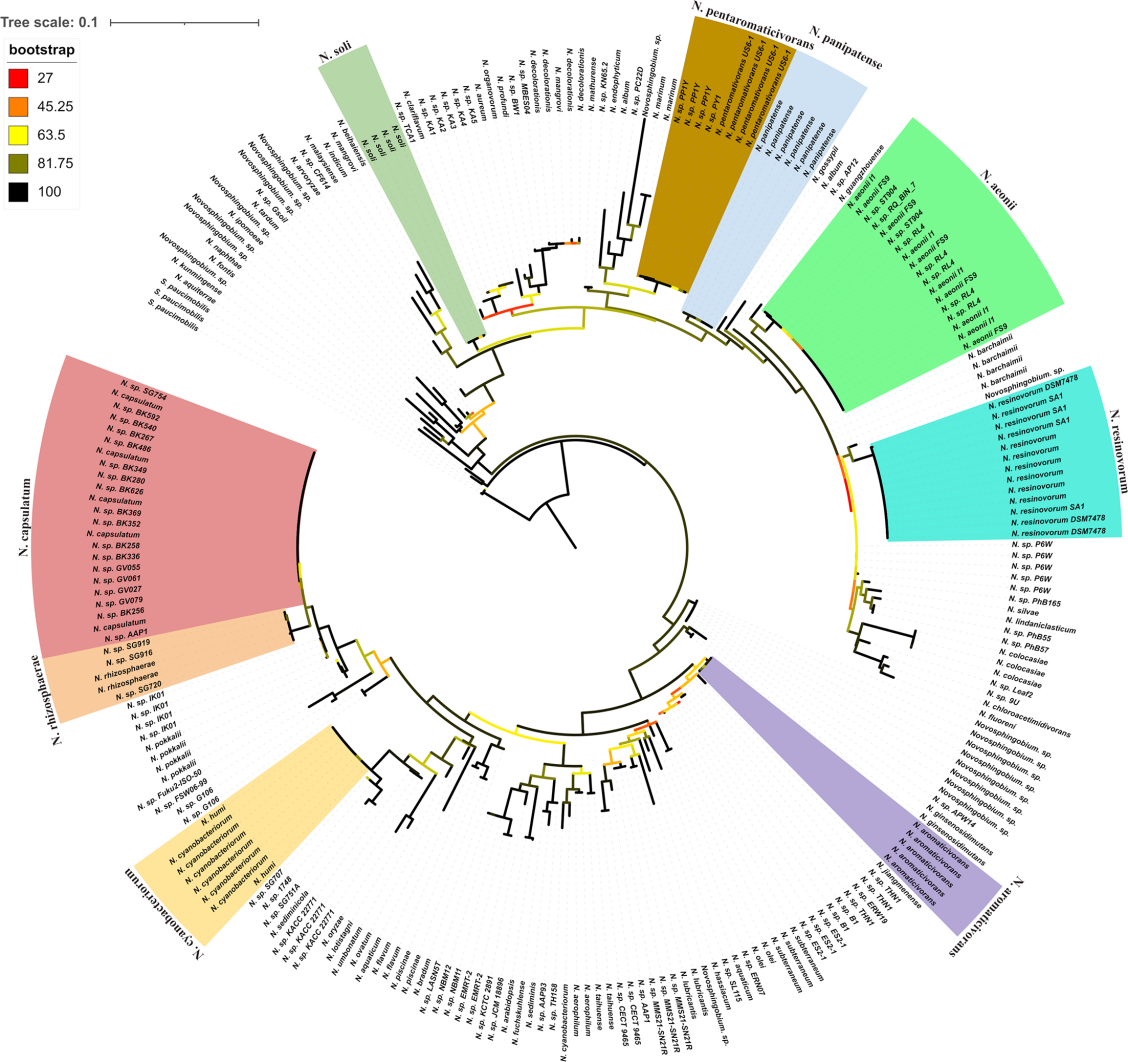
Phylogenetic tree based on 16S rRNA gene sequences of *Novosphingobium* strains. A maximum-likelihood tree was reconstructed using 249 nearly full-length 16S rRNA gene sequences derived from a total of 400 *Novosphingobium* genomes, including 74 type strains and the two novel isolates (I1ᵀ and FS9). *S. paucimobilis* was used as an outgroup. Bootstrap support values are indicated by colours (see legend). Clades corresponding to established *Novosphingobium* species are highlighted with coloured bars, and the proposed *N. aeonii* sp. nov. clade is highlighted in light green. The tree was visualized using the iTOL (v7.0).

Pairwise nucleotide alignments of the nearly full-length 16S rRNA genes (*n*=249) extracted from the assemblies of 398 *Novosphingobium* strains, together with the two isolates (*n*=400), showed that the four additional strains and isolates I1ᵀ and FS9 share >99% sequence similarity with the 16S rRNA gene of the type strain I1ᵀ, strongly supporting their assignment to the same species. Among validly named species, *Novosphingobium barchaimii* LL02ᵀ exhibited the highest 16S rRNA gene sequence similarity to both isolates, with 99.13% identity and 100% sequence coverage for I1^T^ and FS9. Phylogenetic analysis based on 16S rRNA gene sequences further supported this classification. Isolates I1ᵀ and FS9, the two ST904 strains, and strains RQ_Bin_7 and RL4 clustered together in a well-supported and distinct clade, clearly separated from other *Novosphingobium* species (Fig. 3).

## Description of *Novosphingobium aeonii* sp. nov.

*Novosphingobium aeonii* (ae.o’ni.i. N.L. gen. n. *aeonii*, of *Aeonium*, referring to the host plant genus *Aeonium decorum*, from which the type strain was isolated).

Cells are Gram-negative, aerobic, non-sporulating rods, measuring ~1.33–1.95 µm in length and 0.49–0.58 µm in width. Colonies on LB agar are yellow, glossy, circular and smooth with regular margins. Growth occurs at 20–37 °C (optimum 37 °C), at pH 6.0–8.0 (optimum pH 7.0) and in the presence of 0.5–7.8% NaCl (optimum 0.5%). The type strain is oxidase- and catalase-positive. According to API 20NE tests, the type strain assimilates glucose, l-arabinose, d-mannose, *N*-acetyl-glucosamine, d-maltose, malic acid and citrate and exhibits *β*-glucosidase and *β*-galactosidase activities. The major respiratory quinone is Q-10, with minor amounts of Q-9. Polar lipid analysis revealed sphingoglycolipids and several phospholipids, including diphosphatidylglycerol, phosphatidylethanolamine, phosphatidyl-N,N-dimethylethanolamine and phosphatidylglycerol, as well as minor amounts of aminophospholipid, phosphatidylcholine and phosphatidyl-N-monomethylethanolamine. The type strain, I1ᵀ (CECT 31212ᵀ=DSM 119885ᵀ), was isolated from the leaf surface of the succulent plant *Aeonium decorum*. The DNA G+C content of the type strain is 64.76%. The genome consists of four circular replicons, including two plasmids. The complete genome sequences of the type strain are available under GenBank/EMBL/DDBJ accession numbers CP176832–CP176835. Additional strains belonging to this species have been isolated from rhizosphere soils of different plant species and from activated sludge of kraft pulp mill effluents.

## Supplementary material

10.1099/ijsem.0.007220 Supplementary Material 1.

10.1099/ijsem.0.007220 Supplementary Material 2.

10.1099/ijsem.0.007220 Supplementary Material 3.

10.1099/ijsem.0.007220 Supplementary Material 4.

## Abbreviations

ANI: average nucleotide identity
ANI: average nucleotide identity
ANIm: average nucleotide identity based on Mummer
CARD: Comprehensive Antibiotic Resistance Database
COG: Cluster of Orthologous Groups
dDDH: digital DNA–DNA hybridization
dDDH: digital DNA–DNA hybridization
DSMZ: Leibniz Institute–Deutsche Sammlung von Mikroorganismen und Zellkulturen
GGDC: Genome-to-Genome Distance Calculator
ICEs: integrative conjugative elements
iTOL: Interactive Tree of Life
LB: Lysogenic broth
LB: Luria–Bertani
ONT: Oxford Nanopore Technologies
ONT: Oxford Nanopore Technologies
PAHs: polycyclic aromatic hydrocarbons
PM: particulate matter
TYGS: Type (Strain) Genome Server
