# Supplementary material for: Novosphingobium aeonii sp. nov., isolated from leaves of Aeonium decorum, is able to grow with polycyclic aromatic hydrocarbons
Source: Int J Syst Evol Microbiol. 2026 Jun 23;76(6):007220. doi: 10.1099/ijsem.0.007220 (PMC13292080; doi:10.1099/ijsem.0.007220)

Supplementary Information File

***Novosphingobium aeonii* sp. nov., isolated from leaves of *Aeonium decorum,* is able to grow with polycyclic aromatic hydrocarbons**

Ana Segura[1], Lázaro Molina[1], Mafalda Domínguez[1], Félix Velando[1], Irene Hurtado[1], Pieter van Dillewijn[1] and Zulema Udaondo[1,2#]

[1]Environmental Microbiology and Biotechnology, Estación Experimental del Zaidín. CSIC, Granada, Spain

[2]Department of Microbial Biotechnology, Centro Nacional de Biotecnología, CSIC, Madrid, Spain

#Corresponding author: Zulema Udaondo

E-mail: zulema.udaondo@cnb.csic.es

Centro Nacional de Biotecnología, Department of Microbial Biotechnology

28049, Madrid, Spain.

Phone: +34 91 585 45 00

- Index:
  - Supplementary Figures
    - Supplementary Figure 1
    - Supplementary Figure 2
    - Supplementary Figure 3
  - The Type (Strain) Genome Server (TYGS) from the List of Prokaryotic names with Standing in Nomenclature (LPSN) results for I1^{T} isolate.
  - The Type (Strain) Genome Server (TYGS) from the List of Prokaryotic names with Standing in Nomenclature (LPSN) results for the FS9 isolate.
  - Polar lipid and respiratory quinone analyses were carried out by DSMZ Services, Leibniz-Institut DSMZ – Deutsche Sammlung von Mikroorganismen und Zellkulturen GmbH, Braunschweig, Germany.

Supplementary Figures

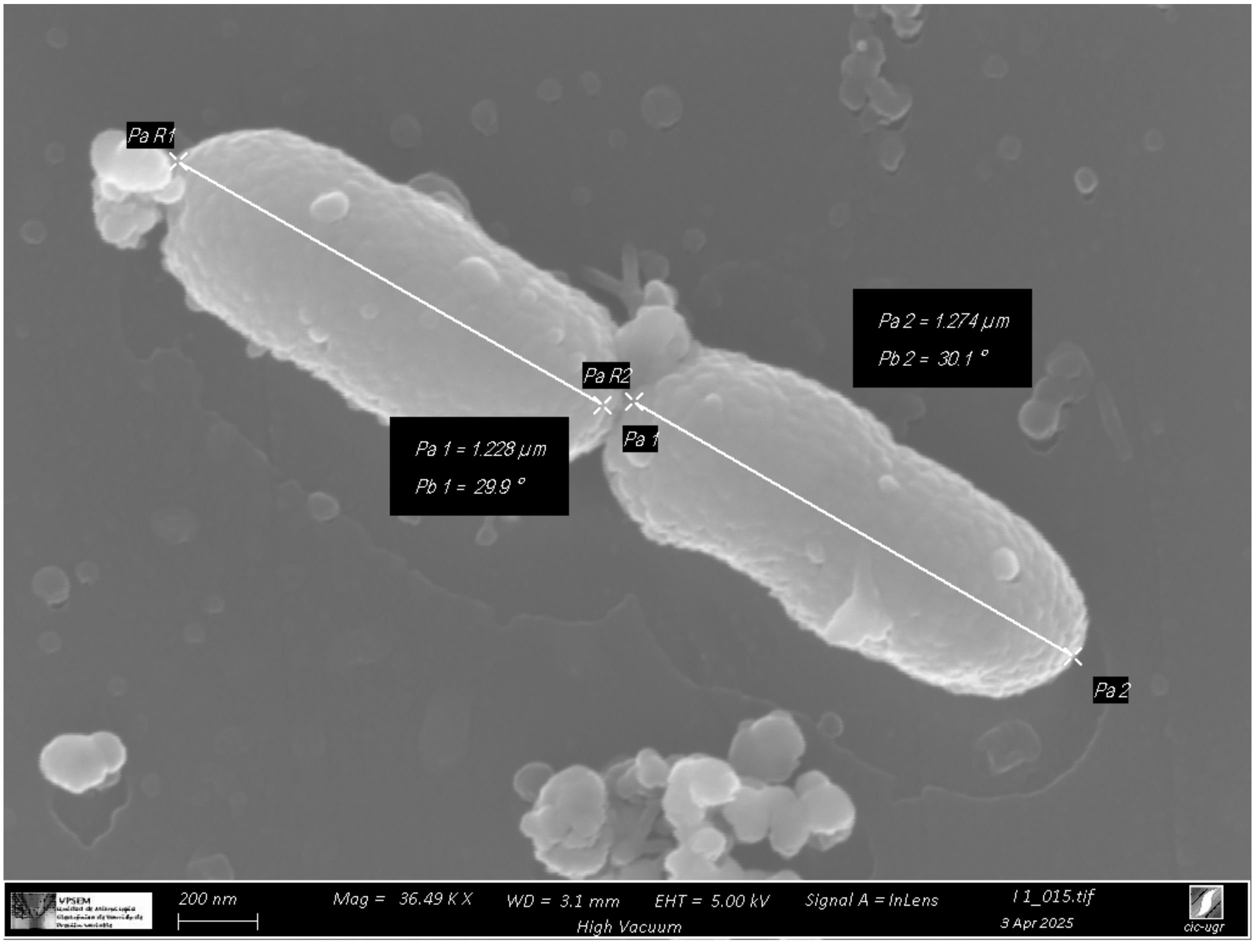

Supplementary Figure 1: Morphology of I1^T isolate grown overnight in Luria-Bertani (LB) liquid medium at 30 °C for 24 hours and observed using scanning electron microscopy. Cells of isolate I1^T are rod-shaped, measuring approximately 1.33 ± 0.15 µm in length and 0.49 ± 0.02 µm in width.

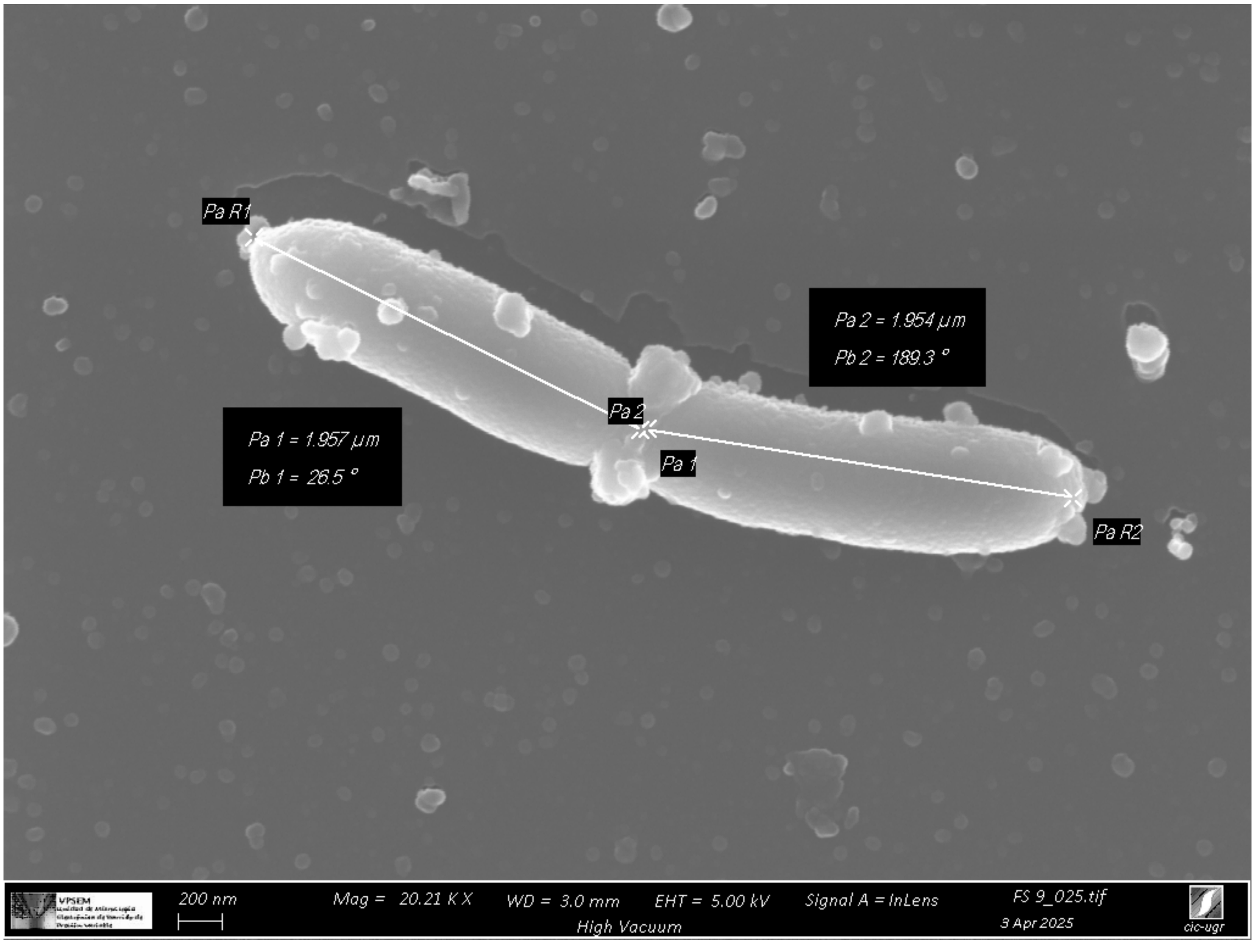

Supplementary Figure 2: Supplementary Figure 1: Morphology of FS9 isolate grown overnight in Luria-Bertani (LB) liquid medium at 30 °C for 24 hours and observed using scanning electron microscopy. Cells of isolate FS9 are rod-shaped, measuring approximately 1.95 ± 0.19 µm in length and 0.58 ± 0.04 µm in width.

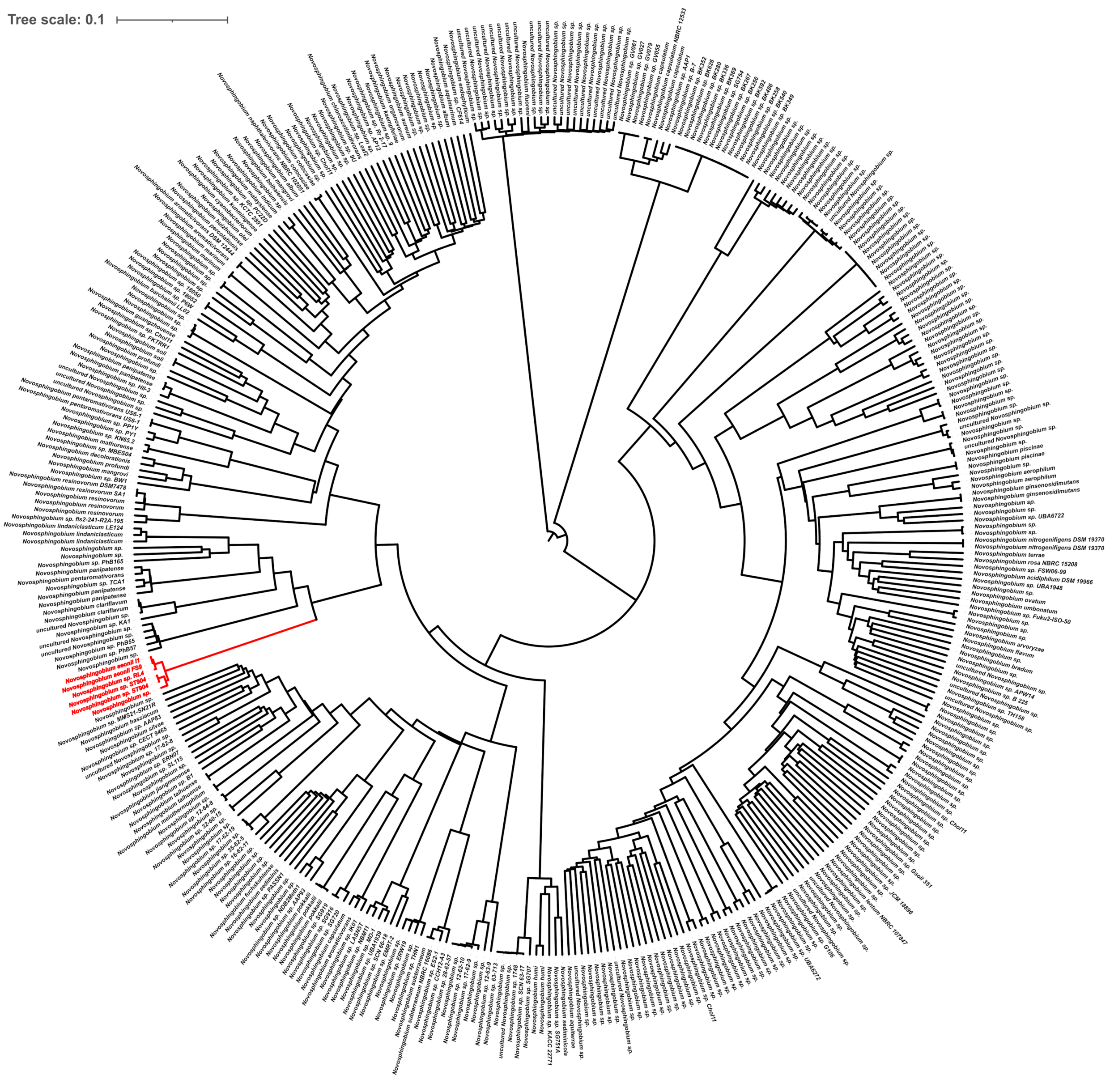

Supplementary Figure 3. ANI-based hierarchical clustering of *Novosphingobium* genomes. Hierarchical clustering based on average nucleotide identity (ANI) values among 400 *Novosphingobium* genomes, including 74 type strains and isolates I1^{T} and FS9. Pairwise ANI values were calculated using ANIm (pyANI) and converted to a distance matrix (1 − ANI). Clustering was performed using Ward's minimum variance method. The proposed *Novosphingobium aeonii* sp. nov. clade, including isolates I1^{T}, FS9, and four additional strains with ANI values ≥95%, forms a well-supported and distinct cluster separated from all currently described species (highlighted in red). The dendrogram was visualized using iTOL v7.0.

PRINT DATE:	2024-12-02 17:23:35 +0100
JOB ID:	8037c80f-d8f5-4110-b0a6-7f6ca30542db
RESULT PAGE:	https://tygs.dsmz.de/user_results/show?guid=8037c80f-d8f5-4110-b0a6-7f6ca30542db

Table 1: Phylogenies

Publication-ready versions of both the genome-scale GBDP tree and the 16S rRNA gene sequence tree can be customized and exported either in SVG (vector graphic) or PNG format from within the phylogeny viewers in your TYGS result page. For publications the **SVG format is recommended** because it is lossless, always keeps its high resolution and can also be easily converted to other popular formats such as PDF or EPS. Please follow the link provided above!

Table 2: Identification

The below list contains the result of the TYGS species identification routine.

Explanation of remarks that might occur in the below table:

remark [R1]: The TYGS type strain database is automatically updated on an almost daily basis. However, if a particular type strain genome is not available in the TYGS database, this can have several reasons which are detailed in the FAQ. You can request an extended 16S rRNA gene analysis via the 16S tree viewer found in your result page to detect **not yet genome-sequenced** type strains relevant for your study.

remark [R2]: > 70% dDDH value (formula d_4) and (almost) minimal dDDH values for gene-content formulae d_0 and d_6 indicate a potentially unreliable identification result and should thus be checked via the 16S rRNA gene sequence similarity. Such strong deviations can, in principle, be caused by sequence contamination.

remark [R3]: G+C content difference of > 1 % indicates a potentially unreliable identification result because within species G+C content varies no more than 1 %, if computed from genome sequences (PMID: 24505073).

Strain	Conclusion	Identification result	Remark
**'I1_corrected'**	potential new species		see [R1]

Table 3: Pairwise comparisons of user genomes vs. type-strain genomes

The following table contains the pairwise dDDH values between your user genomes and the selected type-strain genomes. The dDDH values are provided along with their confidence intervals (C.I.) for the three different GBDP formulas:

• formula d_0 (a.k.a. GGDC formula 1): length of all HSPs divided by total genome length
• formula d_4 (a.k.a. GGDC formula 2): sum of all identities found in HSPs divided by overall HSP length
• formula d_6 (a.k.a. GGDC formula 3): sum of all identities found in HSPs divided by total genome length

Note: Formula d_4 is independent of genome length and is thus robust against the use of incomplete draft genomes. For other reasons for preferring formula d_4, see the FAQ.

Query	Subject	d_0	C.I. d_0	d_4	C.I. d_4	d_6	C.I. d_6	Diff. G+C Percent
**'I1_corrected.fasta'**	*Sphingobium hydrophobicum* CCTCC AB 2015198	14.4	[11.5 - 17.8]	30.6	[28.2 - 33.1]	14.8	[12.3 - 17.6]	1.58
**'I1_corrected.fasta'**	*Sphingobium francense* DSM 26779	14.4	[11.6 - 17.8]	28.9	[26.5 - 31.4]	14.8	[12.3 - 17.6]	0.69
**'I1_corrected.fasta'**	*Sphingobium francense* DSM 26779	14.4	[11.6 - 17.8]	28.8	[26.4 - 31.3]	14.8	[12.3 - 17.6]	0.69
**'I1_corrected.fasta'**	*Novosphingobium lindaniclasticum* DSM 25409	28.7	[25.3 - 32.3]	26.4	[24.0 - 28.9]	27.1	[24.2 - 30.2]	0.21
**'I1_corrected.fasta'**	*Sphingomonas bisphenolicum* AO1	14.5	[11.7 - 17.9]	26.1	[23.8 - 28.6]	14.8	[12.4 - 17.7]	0.71
**'I1_corrected.fasta'**	*Novosphingobium clariflavum* 164	28.9	[25.6 - 32.6]	24.4	[22.1 - 26.9]	26.8	[24.0 - 30.0]	1.02
**'I1_corrected.fasta'**	*Novosphingobium subarcticum* KF1	21.0	[17.8 - 24.6]	24.4	[22.1 - 26.9]	20.5	[17.8 - 23.6]	0.3
**'I1_corrected.fasta'**	*Novosphingobium kaempferiae* Sx8-5T	20.9	[17.7 - 24.5]	22.2	[19.9 - 24.7]	20.2	[17.4 - 23.2]	1.22
**'I1_corrected.fasta'**	*Novosphingobium resinovorum* VKM B-1172	21.6	[18.4 - 25.2]	22.1	[19.8 - 24.5]	20.7	[18.0 - 23.8]	0.69
**'I1_corrected.fasta'**	*Novosphingobium gossypii* DSM 29615	20.2	[17.0 - 23.8]	21.6	[19.4 - 24.1]	19.5	[16.8 - 22.6]	0.13
**'I1_corrected.fasta'**	*Novosphingobium barchaimii* DSM 25411	21.5	[18.2 - 25.1]	21.6	[19.4 - 24.1]	20.6	[17.8 - 23.6]	0.74
**'I1_corrected.fasta'**	*Novosphingobium guangzhouense* DSM 32207	20.1	[16.9 - 23.7]	21.2	[18.9 - 23.6]	19.4	[16.7 - 22.4]	1.26
**'I1_corrected.fasta'**	*Novosphingobium silvae* FGD1	19.4	[16.3 - 23.0]	21.0	[18.8 - 23.4]	18.9	[16.2 - 21.9]	0.32
**'I1_corrected.fasta'**	*Novosphingobium panipatense* SM16T	17.6	[14.5 - 21.1]	20.9	[18.7 - 23.3]	17.3	[14.7 - 20.3]	0.57

Table 4: Strains in your dataset

Joint dataset of automatically determined closest type strains (if this mode was chosen), manually selected type strains (if selected accordingly) and the provided user strains, if provided (marked in yellow).

Strain	Authority	Other deposits	Synonyms	Base pairs	Percent G+C	No. proteins	Goldstamp	Bioproject accession	Biosample accession	Assembly accession	IMG OID
Sphingobium francense DSM 26779	Pal et al. 2005	DSM 16453; CCM 7288; MTCC 6363; Sp+	*Sphingobium francense*	4159 851	65.4	3849		PRJNA520821	SAMN10868428	GCA_004152835	
Novosphingobium panipatense SM16T	Gupta et al. 2009	DSM 22890; CCM 7472; MTCC 9019	*Novosphingobium panipatense*	4079 269	64.2	3917	Gp0157055	PRJEB20707	SAMN06296065	GCA_900182875	
Novosphingobium silvae FGD1	Feng et al. 2020	GDMCC 1.1761; KACC 21283	*Novosphingobium silvae*	4573 238	65.1	4249		PRJNA597879	SAMN13688776	GCA_009856825	
Sphingomonas bisphenolicum AO1	Oshiman et al. 2007		*Sphingomonas bisphenolicum*	5209 012	64.1	5008		PRJDB6608	SAMD00108307	GCA_024349785	
Novosphingobium guangzhouense DSM 32207	Sha et al. 2017 emend. Hördt et al. 2020	GDMCC 1.1110; SA925	*Novosphingobium guangzhouense*	5966 702	63.5	5283	Gp0146782	PRJNA321815	SAMN05004390	GCA_002896965	
Sphingobium hydrophobicum CCTCC AB 2015198	Chen et al. 2016 emend. Hördt et al. 2020	KCTC 42740; C1	*Sphingobium hydrophobicum*	4602 637	63.2	4176	Gp0302338	PRJNA397800	SAMN07488776	GCA_002288285	
Sphingobium francense DSM 26779	Pal et al. 2005	DSM 16453; CCM 7288; MTCC 6363; Sp+	*Sphingobium francense*	4153 308	65.5	3939	Gp0401266	PRJNA546794	SAMN12025198	GCA_013408165	2828378008

Strain	Authority	Other deposits	Synonyms	Base pairs	Percent G+C	No. proteins	Goldstamp	Bioproject accession	Biosample accession	Assembly accession	IMG OID
Novosphingobium subarcticum KF1	(Nohynek et al. 1996) Takeuchi et al. 2001 emend. Hördt et al. 2020	CIP 105288; DSM 10700; JCM 10398; JCM 12332; IFO 16058; NBRC 16058; HAMBI 2110	*Novosphingobium subarcticum*; *Sphingomonas subarctica*	6304486	65.1	5737	Gp0090074	PRJNA239214	SAMN02676962	GCA_000632105	2576861780
Novosphingobium lindaniclasticum DSM 25409	Saxena et al. 2013 emend. Hördt et al. 2020	CCM 7976; LE 124	*Novosphingobium lindaniclasticum*	4857915	64.6	4566	Gp0042293	PRJNA201004	SAMN02471710	GCA_000445125	2558860244
Novosphingobium barchaimii DSM 25411	Niharika et al. 2013 emend. Hördt et al. 2020	CCM 7980; LL02	*Novosphingobium barchaimii*	5307292	64.0	4985	Gp0120795	PRJNA227256	SAMN02727999	GCA_001046635	
Novosphingobium clariflavum 164	Zhang et al. 2017	CICC 11035s; DSM 103351	*Novosphingobium clariflavum*	5198436	65.8	4769		PRJNA893429	SAMN31422749	GCA_026420865	
Novosphingobium kaempferiae Sx8-5T	Sitlaothaworn et al. 2023	TBRC 15600; JCM 35076	*Novosphingobium kaempferiae*	5698134	66.0	5089		PRJNA224116	SAMN23259152	GCF_021227995	
Novosphingobium gossypii DSM 29615	Kämpfer et al. 2015	CIP 110884; CCM 8569; JM-1396; LMG 28605	*Novosphingobium gossypii*	4774106	64.9	4399	Gp0401129				2829944697
Novosphingobium resinovorum VKM B-1172	(Delaporte and Daste 1956) Lim et al. 2007	NCIMB 8767; ATCC 33545; CCUG 33446 A; CCUG 33446 B; CCUG 33446; DSM 7478; LMG 8367; NCIB 8767	*Flavobacterium resinovorum*; *Novosphingobium resinovorum*	5455123	65.4	4944		PRJDB10669	SAMD00253122	GCA_027922145	
I1_corrected.fasta				5761656	64.8	5176					

Methods, Results and References

The genome sequence data were uploaded to the Type (Strain) Genome Server (TYGS), a free bioinformatics platform available under https://tygs.dsmz.de, for a whole genome-based taxonomic analysis [1]. The analysis also made use of recently introduced methodological updates and features [2]. Information on nomenclature, synonymy and associated taxonomic literature was provided by TYGS's sister database, the List of Prokaryotic names with Standing in Nomenclature (LPSN, available at https://lpsn.dsmz.de) [2]. The results were provided by the TYGS on 2024-12-02. The TYGS analysis was subdivided into the following steps:

Determination of closely related type strains

Determination of closest type strain genomes was done in two complementary ways: First, all user genomes were compared against all type strain genomes available in the TYGS database via the MASH algorithm, a fast approximation of intergenomic relatedness [3], and, the ten type strains with the smallest MASH distances chosen per user genome. Second, an additional set of ten closely related type strains was determined via the 16S rDNA gene sequences. These were extracted from the user genomes using RNAmmer [4] and each sequence was subsequently BLASTed [5] against the 16S rDNA gene sequence of each of the currently 22123 type strains available in the TYGS database. This was used as a proxy to find the best 50 matching type strains (according to the bitscore) for each user genome and to subsequently calculate precise distances using the Genome BLAST Distance Phylogeny approach (GBDP) under the algorithm 'coverage' and distance formula d_5 [6]. These distances were finally used to determine the 10 closest type strain genomes for each of the user genomes.

Pairwise comparison of genome sequences

For the phylogenomic inference, all pairwise comparisons among the set of genomes were conducted using GBDP and accurate intergenomic distances inferred under the algorithm 'trimming' and distance formula d_5 [6]. 100 distance replicates were calculated each. Digital DDH values and confidence intervals were calculated using the recommended settings of the GGDC 4.0 [2,6].

Phylogenetic inference

The resulting intergenomic distances were used to infer a balanced minimum evolution tree with branch support via FASTME 2.1.6.1 including SPR postprocessing [7]. Branch support was inferred from 100 pseudo-bootstrap replicates each. The trees were rooted at the midpoint [8] and visualized with PhyD3 [9].

Type-based species and subspecies clustering

The type-based species clustering using a 70% dDDH radius around each of the 14 type strains was done as previously described [1]. The resulting groups are shown in Table 1 and 4. Subspecies clustering was done using a 79% dDDH threshold as previously introduced [10].

Results

Type-based species and subspecies clustering

The resulting species and subspecies clusters are listed in Table 4, whereas the taxonomic identification of the query strains is found in Table 1. Briefly, the clustering yielded 14 species clusters and the provided query strains were assigned to 1 of these. Moreover, user strains were located in 1 of 14 subspecies clusters.

Figure caption SSU tree

Figure 1. Tree inferred with FastME 2.1.6.1 [7] from GBDP distances calculated from 16S rDNA gene sequences. The branch lengths are scaled in terms of GBDP distance formula d_5. The numbers above branches are GBDP pseudo-bootstrap support values > 60 % from 100 replications, with an average branch support of 65.1 %. The tree was rooted at the midpoint [8].

Figure caption genome tree

Figure 2. Tree inferred with FastME 2.1.6.1 [7] from GBDP distances calculated from genome sequences. The branch lengths are scaled in terms of GBDP distance formula d_5. The numbers above branches are GBDP pseudo-bootstrap support values > 60 % from 100 replications, with an average branch support of 66.7 %. The tree was rooted at the midpoint [8].

References

[1] Meier-Kolthoff JP, Göker M. TYGS is an automated high-throughput platform for state-of-the-art genome-based taxonomy. Nat. Commun. 2019;10: 2182. DOI: 10.1038/s41467-019-10210-3

[2] Meier-Kolthoff JP, Sardà Carbasse J, Peinado-Olarte RL, Göker M. TYGS and LPSN: a database tandem for fast and reliable genome-based classification and nomenclature of prokaryotes. Nucleic Acid Res. 2022;50: D801–D807. DOI: 10.1093/nar/gkab902

[3] Ondov BD, Treangen TJ, Melsted P, et al. Mash: Fast genome and metagenome distance estimation using MinHash. Genome Biol 2016;17: 1–14. DOI: 10.1186/s13059-016-0997-x

[4] Lagesen K, Hallin P. RNAmmer: consistent and rapid annotation of ribosomal RNA genes. Nucleic Acids Res. Oxford Univ Press; 2007;35: 3100–3108. DOI: 10.1093/nar/gkm160

[5] Camacho C, Coulouris G, Avagyan V, Ma N, Papadopoulos J, Bealer K, et al. BLAST+: architecture and applications. BMC Bioinformatics. 2009;10: 421. DOI: 10.1186/1471-2105-10-421

[6] Meier-Kolthoff JP, Auch AF, Klenk H-P, Göker M. Genome sequence-based species delimitation with confidence intervals and improved distance functions. BMC Bioinformatics. 2013;14: 60. DOI: 10.1186/1471-2105-14-60

[7] Lefort V, Desper R, Gascuel O. FastME 2.0: A comprehensive, accurate, and fast distance-based phylogeny inference program. Mol Biol Evol. 2015;32: 2798–2800. DOI: 10.1093/molbev/msv150

[8] Farris JS. Estimating phylogenetic trees from distance matrices. Am Nat. 1972;106: 645–667.

[9] Kreft L, Botzki A, Coppens F, Vandepoele K, Van Bel M. PhyD3: A phylogenetic tree viewer with extended phyloXML support for functional genomics data visualization. Bioinformatics. 2017;33: 2946–2947. DOI: 10.1093/bioinformatics/btx324

[10] Meier-Kolthoff JP, Hahnke RL, Petersen J, Scheuner C, Michael V, Fiebig A, et al. Complete genome sequence of DSM 30083^T, the type strain (U5/41^T) of *Escherichia coli*, and a proposal for delineating subspecies in microbial taxonomy. Stand Genomic Sci. 2014;9: 2. DOI: 10.1186/1944-3277-9-2

TYPE (STRAIN) GENOME SERVER

JOB SUMMARY

PRINT DATE: 2024-12-03 09:30:44 +0100

JOB ID: 0f2fbea9-b510-44d8-ab27-6e3353cf956a

RESULT PAGE: https://tygs.dsmz.de/user_results/show?guid=0f2fbea9-b510-44d8-ab27-6e3353cf956a

Table 1: Phylogenies

Publication-ready versions of both the genome-scale GBDP tree and the 16S rRNA gene sequence tree can be customized and exported either in SVG (vector graphic) or PNG format from within the phylogeny viewers in your TYGS result page. For publications the **SVG format is recommended** because it is lossless, always keeps its high resolution and can also be easily converted to other popular formats such as PDF or EPS. Please follow the link provided above!

Table 2: Identification

The below list contains the result of the TYGS species identification routine.

Explanation of remarks that might occur in the below table:

remark [R1]: The TYGS type strain database is automatically updated on an almost daily basis. However, if a particular type strain genome is not available in the TYGS database, this can have several reasons which are detailed in the FAQ. You can request an extended 16S rRNA gene analysis via the 16S tree viewer found in your result page to detect **not yet genome-sequenced** type strains relevant for your study.

remark [R2]: > 70% dDDH value (formula d_4) and (almost) minimal dDDH values for gene-content formulae d_0 and d_6 indicate a potentially unreliable identification result and should thus be checked via the 16S rRNA gene sequence similarity. Such strong deviations can, in principle, be caused by sequence contamination.

remark [R3]: G+C content difference of > 1 % indicates a potentially unreliable identification result because within species G+C content varies no more than 1 %, if computed from genome sequences (PMID: 24505073).

Strain	Conclusion	Identification result	Remark
**'FS9_corrected'**	potential new species		see [R1]

Table 3: Pairwise comparisons of user genomes vs. type-strain genomes

The following table contains the pairwise dDDH values between your user genomes and the selected type-strain genomes. The dDDH values are provided along with their confidence intervals (C.I.) for the three different GBDP formulas:

• formula d_0 (a.k.a. GGDC formula 1): length of all HSPs divided by total genome length
• formula d_4 (a.k.a. GGDC formula 2): sum of all identities found in HSPs divided by overall HSP length
• formula d_6 (a.k.a. GGDC formula 3): sum of all identities found in HSPs divided by total genome length

Note: Formula d_4 is independent of genome length and is thus robust against the use of incomplete draft genomes. For other reasons for preferring formula d_4, see the FAQ.

Query	Subject	d_0	C.I. d_0	d_4	C.I. d_4	d_6	C.I. d_6	Diff. G+C Percent
**'FS9_corrected.fasta'**	*Sphingobium hydrophobicum* CCTCC AB 2015198	14.4	[11.5 - 17.8]	30.5	[28.1 - 33.0]	14.8	[12.3 - 17.6]	1.48
**'FS9_corrected.fasta'**	*Sphingobium francense* DSM 26779	14.4	[11.6 - 17.8]	28.9	[26.5 - 31.4]	14.8	[12.3 - 17.6]	0.79
**'FS9_corrected.fasta'**	*Sphingobium francense* DSM 26779	14.4	[11.6 - 17.8]	28.8	[26.4 - 31.3]	14.8	[12.3 - 17.6]	0.78
**'FS9_corrected.fasta'**	*Novosphingobium lindaniclasticum* DSM 25409	28.3	[24.9 - 31.9]	26.4	[24.0 - 28.9]	26.7	[23.8 - 29.8]	0.12
**'FS9_corrected.fasta'**	*Sphingomonas bisphenolicum* AO1	14.5	[11.7 - 17.9]	26.1	[23.8 - 28.6]	14.8	[12.3 - 17.7]	0.62
**'FS9_corrected.fasta'**	*Novosphingobium subarcticum* KF1	21.1	[17.8 - 24.7]	24.4	[22.1 - 26.9]	20.6	[17.8 - 23.6]	0.39
**'FS9_corrected.fasta'**	*Novosphingobium clariflavum* 164	28.5	[25.2 - 32.1]	24.4	[22.1 - 26.9]	26.5	[23.6 - 29.6]	1.11
**'FS9_corrected.fasta'**	*Novosphingobium soli* CCM 7706	20.3	[17.1 - 23.9]	22.5	[20.2 - 25.0]	19.7	[17.0 - 22.8]	3.47
**'FS9_corrected.fasta'**	*Novosphingobium kaempferiae* Sx8-5T	20.7	[17.5 - 24.4]	22.2	[19.9 - 24.7]	20.0	[17.3 - 23.1]	1.32
**'FS9_corrected.fasta'**	*Novosphingobium resinovorum* VKM B-1172	21.4	[18.2 - 25.0]	22.1	[19.8 - 24.5]	20.6	[17.8 - 23.6]	0.78
**'FS9_corrected.fasta'**	*Novosphingobium panipatense* SM16T	18.0	[14.9 - 21.5]	21.7	[19.5 - 24.2]	17.7	[15.1 - 20.7]	0.48
**'FS9_corrected.fasta'**	*Novosphingobium gossypii* DSM 29615	20.0	[16.8 - 23.6]	21.6	[19.4 - 24.1]	19.4	[16.7 - 22.4]	0.23
**'FS9_corrected.fasta'**	*Novosphingobium barchaimii* DSM 25411	21.3	[18.1 - 24.9]	21.6	[19.4 - 24.1]	20.4	[17.7 - 23.5]	0.65
**'FS9_corrected.fasta'**	*Novosphingobium guangzhouense* DSM 32207	20.1	[16.9 - 23.7]	21.2	[18.9 - 23.6]	19.4	[16.7 - 22.4]	1.17
**'FS9_corrected.fasta'**	*Novosphingobium silvae* FGD1	19.3	[16.2 - 22.9]	21.0	[18.8 - 23.4]	18.8	[16.1 - 21.8]	0.42

Table 4: Strains in your dataset

Joint dataset of automatically determined closest type strains (if this mode was chosen), manually selected type strains (if selected accordingly) and the provided user strains, if provided (marked in yellow).

Strain	Authority	Other deposits	Synonyms	Base pairs	Percent G+C	No. proteins	Goldstamp	Bioproject accession	Biosample accession	Assembly accession	IMG OID
Sphingobium francense DSM 26779	Pal et al. 2005	DSM 16453; CCM 7288; MTCC 6363; Sp+	*Sphingobium francense*	4159 851	65.4	3849		PRJNA520821	SAMN10868428	GCA_004152835	
Novosphingobium panipatense SM16T	Gupta et al. 2009	DSM 22890; CCM 7472; MTCC 9019	*Novosphingobium panipatense*	4079 269	64.2	3917	Gp0157055	PRJEB20707	SAMN06296065	GCA_900182875	
Novosphingobium silvae FGD1	Feng et al. 2020	GDMCC 1.1761; KACC 21283	*Novosphingobium silvae*	4573 238	65.1	4249		PRJNA597879	SAMN13688776	GCA_009856825	
Sphingomonas bisphenolicum AO1	Oshiman et al. 2007		*Sphingomonas bisphenolicum*	5209 012	64.1	5008		PRJDB6608	SAMD00108307	GCA_024349785	
Novosphingobium guangzhouense DSM 32207	Sha et al. 2017 emend. Hördt et al. 2020	GDMCC 1.1110; SA925	*Novosphingobium guangzhouense*	5966 702	63.5	5283	Gp0146782	PRJNA321815	SAMN05004390	GCA_002896965	
Sphingobium hydrophobicum CCTCC AB 2015198	Chen et al. 2016 emend. Hördt et al. 2020	KCTC 42740; C1	*Sphingobium hydrophobicum*	4602 637	63.2	4176	Gp0302338	PRJNA397800	SAMN07488776	GCA_002288285	
Sphingobium francense DSM 26779	Pal et al. 2005	DSM 16453; CCM 7288; MTCC 6363; Sp+	*Sphingobium francense*	4153 308	65.5	3939	Gp0401266	PRJNA546794	SAMN12025198	GCA_013408165	2828378008

Strain	Authority	Other deposits	Synonyms	Base pairs	Percent G+C	No. proteins	Goldstamp	Bioproject accession	Biosample accession	Assembly accession	IMG OID
Novosphingobium subarcticum KF1	(Nohynek et al. 1996) Takeuchi et al. 2001 emend. Hördt et al. 2020	CIP 105288; DSM 10700; JCM 10398; JCM 12332; IFO 16058; NBRC 16058; HAMBI 2110	*Novosphingobium subarcticum*; *Sphingomonas subarctica*	6304486	65.1	5737	Gp0090074	PRJNA239214	SAMN02676962	GCA_000632105	2576861780
Novosphingobium lindaniclasticum DSM 25409	Saxena et al. 2013 emend. Hördt et al. 2020	CCM 7976; LE 124	*Novosphingobium lindaniclasticum*	4857915	64.6	4566	Gp0042293	PRJNA201004	SAMN02471710	GCA_000445125	2558860244
Novosphingobium barchaimii DSM 25411	Niharika et al. 2013 emend. Hördt et al. 2020	CCM 7980; LL02	*Novosphingobium barchaimii*	5307292	64.0	4985	Gp0120795	PRJNA227256	SAMN02727999	GCA_001046635	
Novosphingobium soli CCM 7706	Kämpfer et al. 2011	CCUG 58493; DSM 22821; CC-TPE-1	*Novosphingobium soli*	4474282	68.1	4130		PRJNA595761	SAMN43281619	GCA_042432915	
Novosphingobium clariflavum 164	Zhang et al. 2017	CICC 11035s; DSM 103351	*Novosphingobium clariflavum*	5198436	65.8	4769		PRJNA893429	SAMN31422749	GCA_026420865	
Novosphingobium kaempferiae Sx8-5T	Sitlaothaworn et al. 2023	TBRC 15600; JCM 35076	*Novosphingobium kaempferiae*	5698134	66.0	5089		PRJNA224116	SAMN23259152	GCF_021227995	
Novosphingobium gossypii DSM 29615	Kämpfer et al. 2015	CIP 110884; CCM 8569; JM-1396; LMG 28605	*Novosphingobium gossypii*	4774106	64.9	4399	Gp0401129				2829944697

Strain	Authority	Other deposits	Synonyms	Base pairs	Percent G+C	No. proteins	Goldstamp	Bioproject accession	Biosample accession	Assembly accession	IMG OID
Novosphingobium resinovorum VKM B-1172	(Delaporte and Daste 1956) Lim et al. 2007	NCIMB 8767; ATCC 33545; CCUG 33446 A; CCUG 33446 B; CCUG 33446; DSM 7478; LMG 8367; NCIB 8767	*Flavobacterium resinovorum*; *Novosphingobium resinovorum*	5455123	65.4	4944		PRJDB10669	SAMD00253122	GCA_027922145	
FS9_corrected.fasta				5920010	64.7	5319					

Methods, Results and References

The genome sequence data were uploaded to the Type (Strain) Genome Server (TYGS), a free bioinformatics platform available under https://tygs.dsmz.de, for a whole genome-based taxonomic analysis [1]. The analysis also made use of recently introduced methodological updates and features [2]. Information on nomenclature, synonymy and associated taxonomic literature was provided by TYGS's sister database, the List of Prokaryotic names with Standing in Nomenclature (LPSN, available at https://lpsn.dsmz.de) [2]. The results were provided by the TYGS on 2024-12-02. The TYGS analysis was subdivided into the following steps:

Determination of closely related type strains

Determination of closest type strain genomes was done in two complementary ways: First, all user genomes were compared against all type strain genomes available in the TYGS database via the MASH algorithm, a fast approximation of intergenomic relatedness [3], and, the ten type strains with the smallest MASH distances chosen per user genome. Second, an additional set of ten closely related type strains was determined via the 16S rDNA gene sequences. These were extracted from the user genomes using RNAmmer [4] and each sequence was subsequently BLASTed [5] against the 16S rDNA gene sequence of each of the currently 22123 type strains available in the TYGS database. This was used as a proxy to find the best 50 matching type strains (according to the bitscore) for each user genome and to subsequently calculate precise distances using the Genome BLAST Distance Phylogeny approach (GBDP) under the algorithm 'coverage' and distance formula d_5 [6]. These distances were finally used to determine the 10 closest type strain genomes for each of the user genomes.

Pairwise comparison of genome sequences

For the phylogenomic inference, all pairwise comparisons among the set of genomes were conducted using GBDP and accurate intergenomic distances inferred under the algorithm 'trimming' and distance formula d_5 [6]. 100 distance replicates were calculated each. Digital DDH values and confidence intervals were calculated using the recommended settings of the GGDC 4.0 [2,6].

Phylogenetic inference

The resulting intergenomic distances were used to infer a balanced minimum evolution tree with branch support via FASTME 2.1.6.1 including SPR postprocessing [7]. Branch support was inferred from 100 pseudo-bootstrap replicates each. The trees were rooted at the midpoint [8] and visualized with PhyD3 [9].

Type-based species and subspecies clustering

The type-based species clustering using a 70% dDDH radius around each of the 15 type strains was done as previously described [1]. The resulting groups are shown in Table 1 and 4. Subspecies clustering was done using a 79% dDDH threshold as previously introduced [10].

Results

Type-based species and subspecies clustering

The resulting species and subspecies clusters are listed in Table 4, whereas the taxonomic identification of the query strains is found in Table 1. Briefly, the clustering yielded 15 species clusters and the provided query strains were assigned to 1 of these. Moreover, user strains were located in 1 of 15 subspecies clusters.

Figure caption SSU tree

Figure 1. Tree inferred with FastME 2.1.6.1 [7] from GBDP distances calculated from 16S rDNA gene sequences. The branch lengths are scaled in terms of GBDP distance formula d_5. The numbers above branches are GBDP pseudo-bootstrap support values > 60 % from 100 replications, with an average branch support of 59.2 %. The tree was rooted at the midpoint [8].

Figure caption genome tree

Figure 2. Tree inferred with FastME 2.1.6.1 [7] from GBDP distances calculated from genome sequences. The branch lengths are scaled in terms of GBDP distance formula d_5. The numbers above branches are GBDP pseudo-bootstrap support values > 60 % from 100 replications, with an average branch support of 65.1 %. The tree was rooted at the midpoint [8].

References

[1] Meier-Kolthoff JP, Göker M. TYGS is an automated high-throughput platform for state-of-the-art genome-based taxonomy. Nat. Commun. 2019;10: 2182. DOI: 10.1038/s41467-019-10210-3

[2] Meier-Kolthoff JP, Sardà Carbasse J, Peinado-Olarte RL, Göker M. TYGS and LPSN: a database tandem for fast and reliable genome-based classification and nomenclature of prokaryotes. Nucleic Acid Res. 2022;50: D801–D807. DOI: 10.1093/nar/gkab902

[3] Ondov BD, Treangen TJ, Melsted P, et al. Mash: Fast genome and metagenome distance estimation using MinHash. Genome Biol 2016;17: 1–14. DOI: 10.1186/s13059-016-0997-x

[4] Lagesen K, Hallin P. RNAmmer: consistent and rapid annotation of ribosomal RNA genes. Nucleic Acids Res. Oxford Univ Press; 2007;35: 3100–3108. DOI: 10.1093/nar/gkm160

[5] Camacho C, Coulouris G, Avagyan V, Ma N, Papadopoulos J, Bealer K, et al. BLAST+: architecture and applications. BMC Bioinformatics. 2009;10: 421. DOI: 10.1186/1471-2105-10-421

[6] Meier-Kolthoff JP, Auch AF, Klenk H-P, Göker M. Genome sequence-based species delimitation with confidence intervals and improved distance functions. BMC Bioinformatics. 2013;14: 60. DOI: 10.1186/1471-2105-14-60

[7] Lefort V, Desper R, Gascuel O. FastME 2.0: A comprehensive, accurate, and fast distance-based phylogeny inference program. Mol Biol Evol. 2015;32: 2798–2800. DOI: 10.1093/molbev/msv150

[8] Farris JS. Estimating phylogenetic trees from distance matrices. Am Nat. 1972;106: 645–667.

[9] Kreft L, Botzki A, Coppens F, Vandepoele K, Van Bel M. PhyD3: A phylogenetic tree viewer with extended phyloXML support for functional genomics data visualization. Bioinformatics. 2017;33: 2946–2947. DOI: 10.1093/bioinformatics/btx324

[10] Meier-Kolthoff JP, Hahnke RL, Petersen J, Scheuner C, Michael V, Fiebig A, et al. Complete genome sequence of DSM 30083^T, the type strain (U5/41^T) of *Escherichia coli*, and a proposal for delineating subspecies in microbial taxonomy. Stand Genomic Sci. 2014;9: 2. DOI: 10.1186/1944-3277-9-2

DSMZ Services polar lipid report

DPG = Diphosphatidylglycerol

PC = Phosphatidylcholine

PE = Phosphatidylethanolamine

PME = Phosphatidyl-N-monomethylethanolamine

PDME = Phosphatidyl-N,N-dimethylethanolamine

PG = Phosphatidylglycerol

APL = Aminophospholipid

SGL = Sphingoglycolipid

L = Lipid

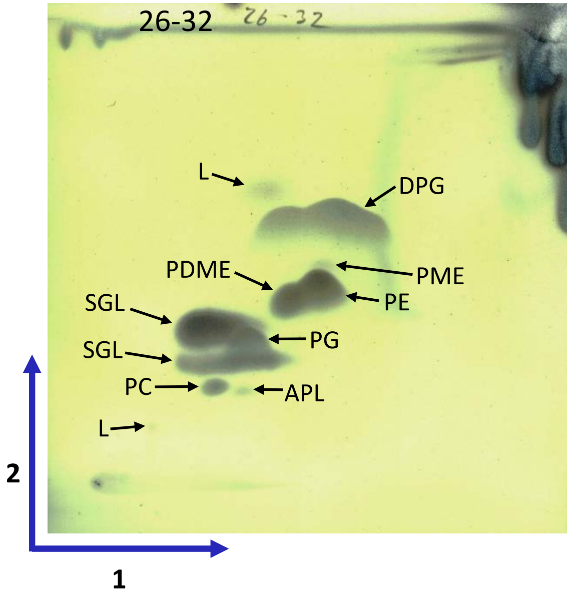

Supplement: Supplementary Material 1. [file ijsem-76-07220-s001.pdf]
